# NSUN2 Promotes Tumor Progression and Regulates Immune Infiltration in Nasopharyngeal Carcinoma

**DOI:** 10.3389/fonc.2022.788801

**Published:** 2022-04-29

**Authors:** Xinya Tong, Yilan Xiang, Yuanbo Hu, Yingying Hu, He Li, Huilin Wang, Kong-Nan Zhao, Xiangyang Xue, Shanli Zhu

**Affiliations:** ^1^ Wenzhou Collaborative Innovation Center of Gastrointestinal Cancer in Basic Research and Precision Medicine, Wenzhou Key Laboratory of Cancer-related Pathogens and Immunity, Department of Microbiology and Immunology, Institute of Molecular Virology and Immunology, School of Basic Medical Sciences, Wenzhou Medical University, Wenzhou, China; ^2^ Department of Radiology, the First Affiliated Hospital of Wenzhou Medical University, Wenzhou, China; ^3^ Department of Gastrointestinal Surgery, Second Affiliated Hospital and Yuying Children’s Hospital of Wenzhou Medical University, Wenzhou, China; ^4^ Department of Obstetrics and Gynecology, The Second Affiliated Hospital and Yuying Children’s Hospital of Wenzhou Medical University, Wenzhou, China; ^5^ Department of Otolaryngology-Head and Neck Surgery, First Affiliated Hospital of Wenzhou Medical University, Wenzhou, China

**Keywords:** nasopharyngeal carcinoma, NSUN2, oncogene, tumor immune microenvironment, immune infiltration

## Abstract

Nasopharyngeal carcinoma (NPC) is one of the most common malignancies in the head and neck with a complex etiology, such as environmental factors, genetic factors, and Epstein–Barr virus infection. The NOP2/Sun domain family, member 2 (NSUN2) is a methyltransferase of m5C methylation modification that has been reported to be involved in the occurrence and progression of various tumors, but its role in NPC remains unclear. In this study, we found that NSUN2 was upregulated in NPC and predicted a poor prognosis for NPC patients in both GEO datasets and our tissue microarrays containing 125 NPC tissues. Next, we demonstrated that NSUN2 promoted the proliferation, migration, and invasion of NPC cells *in vitro*. Additionally, the differential expression genes between NSUN2-high and low expression patients were mainly enriched in multi-immune cell activation and proliferation. Furthermore, NSUN2 negatively regulates immune cell infiltration in the tumor microenvironment (TME) of NPC, which indicates that the NSUN2 level may be negatively correlated with the sensitivity of immunotherapy and chemotherapy. In conclusion, our findings highlight that NSUN2 might act as an important oncogene involved in NPC progression and serve as a potential biomarker to predict poor prognosis and drug sensitivity of NPC patients.

## Introduction

Nasopharyngeal carcinoma (NPC) is a malignancy that occurs in the nasopharyngeal epithelium and is highly metastatic and aggressive (1). It is very common in Southeast Asia and southern China, and its incidence is closely related to Epstein–Barr virus (EBV) infection, genes, race, and environmental factors (2).The current treatment is a combination of radiotherapy and chemotherapy, which has greatly improved the prognosis of NPC patients. However, the 5-year survival rate for patients with advanced nasopharyngeal cancer is still very low (1, 3). Therefore, exploring the pathogenesis of NPC and finding novel biomarkers plays a crucial role in finding appropriate treatment strategies.

RNA modification plays an indispensable role in the occurrence and development of malignancy. Currently, more than 100 RNA modifications have been reported (4). Among them, 5-methylcytosine (m5C), one of the most important types of post-transcriptional modifications, was first found in tRNAs and rRNA, and has recently been found in mRNA and non-coding RNA (5). Similar to the N6-methyladenosine (m6A) modification, the m5C modification has its own methyltransferase “Writer,” demethylase “Eraser,” and binding protein “Reader” (5, 6). NSUN2, a methyltransferase of m5C, is responsible for the m5C modification in the mRNA of mammals (7). Currently, published data have shown that NSUN2 is overexpressed in various tumors, such as breast cancer, colorectal cancer, and gallbladder carcinoma, and is associated with a series of malignant phenotypes such as tumor proliferation and migration (8–10). NSUN2 and its reading protein Y-box binding protein 1 (YBX-1) are highly expressed in human urothelial carcinoma of the bladder (UCB) and act as oncogenes in an m5C-dependent manner, promoting the development of UCB by increasing the mRNA stability of heparin-binding growth factor (HDGF) (7, 11). NSUN2 inhibits the translation of p27 by methylating the 5’-untranslated region (UTR) region of p27 mRNA. Overexpression of NSUN2 can reduce the expression of p27 and increase the level of cyclin‐dependent kinase 1 (CDK1), thus promoting cell proliferation in human diploid fibroblasts (12). NSUN2 upregulates the TEAD1 expression in an m5C mediated methylation-promoted proliferation and migration of hypopharyngeal squamous cell carcinoma cells (13). A team headed by Lu has reported that NSUN2 expression was elevated in squamous cells of head and neck carcinoma (HNSC), including NPC (14). Another study showed that NSUN2 expression was negatively associated with T-cell activation and its expression level might act as a potential immunotherapy marker in HNSC (15). However, few studies have focused on the biological role of NSUN2 in NPC.

In this study, we used the three data entities of the Gene Expression Omnibus (GEO) database to identify the expression levels of 10 m5C regulators in NPC and found that NSUN2 was generally upregulated in NPC tissues and predicted a poor prognosis for NPC patients. Next, we analyzed the expression level of NSUN2, clinicopathological characteristics, and prognosis in tissue microarrays (TMA) containing 125 NPC tissues by immunohistochemistry (IHC). Additionally, we demonstrated that NSUN2 could promote the proliferation, migration, and invasion of NPC cells *in vitro*. Furthermore, based on the GEO database and bioinformatics analysis, we found that the expression level of NSUN2 was negatively associated with the degree of immune cell infiltration, immune checkpoint blockades (ICBs), and chemotherapeutic sensitivity in NPC. Together, our study identified that NSUN2 might serve as a predictive biomarker for poor prognosis and drug sensitivity of NPC patients and may play a vital role in NPC progression.

## Materials and Methods

### NPC Dataset Source and Bioinformation Analysis

The raw data and corresponding clinical information were sourced from the Gene Expression Omnibus Database (GEO) (https://www.ncbi.nlm.nih.gov/geo/). The expression array data (GSE53819) contained 18 NPC tissues and 18 non-cancerous nasopharyngeal tissues, GSE12452 contained 31 NPC tissues and 10 non-cancerous nasopharyngeal tissues, and GSE61218 contained 10 NPC tissues and 6 non-cancerous nasopharyngeal tissues. The high throughput sequencing data GSE102349 containing 113 NPC tissues and corresponding progress-free survival (PFS) information were used for subsequent analysis.

### Identification of Differentially Expressed m5C-Related Regulators in NPC

Ten m5C-related regulators were selected for differential expression analysis. Differential expression of m5C RNA methylation regulatory factors in NPC was performed using the Wilcoxon test from GSE53819 and GSE12452. *P  <0.05, **P <0.01, and ***P <0.001 are significant.

### Survival Analysis

The R packages survival and survminer were used for the survival analysis. A total of 88 out of 113 patients from GSE102349 with detailed progress-free survival information were included for survival analysis. A Kaplan–Meier analysis was used to plot the survival curve, and log rank was used as the statistical significance test.

### Immunohistochemistry (IHC)

The TMA with 125 NPC patients were purchased from Superbiotek (Shanghai, China). IHC determined the expression of NSUN2 in NPC and normal tissues as previously described (16). After deparaffinizing and blocking endogenous peroxidase, the slides were incubated with antibody against NSUN2 (1:800 dilution, 20854-1-AP, Proteintech, Wuhan, China). Arrays were then washed with PBS and incubated for 30 min with the EnVision™+ Dual Link System-HRP (Dako, Carpinteria, CA). After rinsing thrice with PBS for 3 min each time, the slides were incubated with DAB reagent (Dako, Carpinteria, CA) for 3–5 min and evaluated under a light microscope. The TMA were then counter-stained with hematoxylin and observed under a Leica microscope (DM4000b, Wetzlar, Germany). The results were evaluated independently by two blinded pathologists according to the following scoring criteria: 0, negative; 1, weak; and 2–3, strong. The degree of staining was scored as the proportion of the positive staining area relative to the whole cancerous area: 0, <5%; 1, 5–25%; 2, 26–50%; 3, 51–75%; and 4, >75%.

### Reagents and Cell Culture

Human NPC cell lines C666-1 and CNE were purchased from the Chinese Academy of Medical Sciences Cell Bank (Shanghai, China). Both were cultured in RPMI-1640 (Gibco, Thermo Fisher Scientific) medium supplemented with 10% fetal bovine serum (FBS, Gibco) at 37°C.

### Plasmid and siRNA Transfection

The full-length coding sequence (CDS) of NSUN2 with an HA tag was subcloned into the EcoR I and XhoI restriction sites of the pcDNA3.1(+) vector. The construct was confirmed by DNA sequencing. RiboBio (Guangzhou, China) synthesized the small interfering RNA (siRNA) against NSUN2 (si-NSUN2-1,5’‐GAAGCATCGTGCTGAAGTA‐3’; si-NSUN2-2,5’‐GGGTTATCCTCACAAATGA‐3’). Plasmids and siRNA transfection was performed using Lipofectamine 2000 (Invitrogen Life Technologies^®^, Carlsbad, Calif., USA) according to the instructions. The expression level of NSUN2 was tested by Western blot with anti-NSUN2 and anti-HA-Tag (#2367, Cell Signaling Technology (CST), Danvers, MA, USA) antibodies, respectively.

### Western Blot

Total proteins were extracted from the transfected cells with RIPA lysis buffer (Beyotime, Haimen, China) containing a protease and phosphatase inhibitor mixture. The proteins were separated by sodium dodecyl sulfate-polyacrylamide gel (SDS-PAGE), transferred to a polyvinylidene fluoride (PVDF) membrane (Bio-Rad, Hercules, CA) and blocked with 5% skim milk at room temperature for 1 h. The membranes were respectively incubated with primary mouse antibody against-HA (1:1,000 dilution), rabbit anti-NSUN2 (1:1,000 dilution), and mouse anti-GAPDH (1:1,000 dilution, AB-M-M001, Good Here, Hangzhou, China) overnight at 4°C. After washing thrice with TBST, the membranes were incubated with secondary antibodies (HRP-conjugated goat anti-rabbit IgG or HRP-conjugated goat anti-mouse IgG) at room temperature for 1 h. After three washes with TBST, the protein bands were visualized using enhanced chemiluminescence detection reagents.

### Cell Proliferation and Clone Formation Assays

Cell proliferation was performed by using a Cell Counting Kit‐8 (CCK8) and clone formation experiments. The NPC cell lines, C666-1 and CNE, were transfected with siRNA or pcDNA3.1(+)/NSUN2 plasmid. After 48 h, 3,000/well transfected cells were seeded into a 96-well plate in quintuplicate. After 1, 2, 3, and 4 days, the medium was discarded and replaced with fresh complete medium with 10% CCK8 (Solarbio Science & Technology Co., Ltd., Beijing, China) solution that was added to each well. After incubation at 37 °C for 3 h, the OD value was measured at 450 nm. For the colony formation, transfected cells were evenly spread in a 6-well plate (300/well), and the medium was changed every four days for 10–14 days. After removing the medium, the cells were washed twice with PBS, fixed with 4% paraformaldehyde for 15 min and stained with 0.1% crystal violet (Beyotime Biotechnology, Shanghai, China) for 15 min. The number of colonies was counted under a microscope. The experiments were repeated three times.

### Transwell Migration and Invasion Analysis

The Transwell system was used to assess the ability of cells to migrate and invade. In the migration assay, the cells were seeded in a 6-well plate overnight and transfected with siRNA or NSUN2 plasmid, respectively. After 24 h, the cells were trypsinized and 1 × 10^5^ transfected cells suspended in 200 μl of serum-free medium were seeded in the upper chamber, and 600 μl of complete medium with 10% FBS was added to the lower chamber. After 28 h (C666-1) or 24 h (CNE), the upper chambers with residual cells were removed, and the cells under the surface were fixed with 4% paraformaldehyde for 15 min, and then stained with 0.1% crystal violet for 15 min. Five areas were randomly selected for counting under a microscope (Leica, London, UK). In the invasion assay, the chambers were coated with Matrigel (BD Pharringen, San Jose, CA). Similar protocols were followed as for the above-mentioned migration assay.

### Generation of Differential Expression Genes (DEGs) Between NSUN2-High and Low Expression Groups

A total of 88 patients from GSE102349 were divided into NSUN2-high expression and low expression groups based on the survival analysis. Package limma was used to determine the DEGs between the two groups. DEGs with |log2FC| >0.5 and a false discovery rate (FDR) <0.05 were considered significant.

### Functional Enrichment Analysis of the DEGs Between the NSUN2-High and Low Expression Groups

GO and KEGG analyses were performed using the package cluster Profiler, enrichplot, and ggplot2. Only the terms with a *P*-value of <0.05 were considered significantly enriched.

### Estimation of Immune Infiltration of NPC

Immune and stromal scores were calculated by applying the ESTIMATE algorithm, which can reveal the enrichment of stromal and immune cell gene signatures. The microenvironment cell population-counter (MCP counter) and single-sample GSEA (ssGSEA) algorithm were performed to evaluate the absolute abundance of immune and stromal cells. The enrichment score of 16 immune cells was analyzed using the R package GSVA.

### Chemotherapeutic Response Prediction

The R package pRRophetic was used to estimate the chemotherapeutic response determined by the half maximal inhibitory concentration (IC50) of each NPC patient on the Genomics of Drug Sensitivity in Cancer (GDSC) website (17, 18).

### Statistical Analysis

All statistical analyses were performed using the R software (version 4.0.2), GraphPad Prism 8.0, or SPSS 20.0 software (SPSS, version 20.0, IBM, New York, USA). Data are presented as mean ± standard deviation (SD) or percentage (%). Comparisons between two groups were made using a two-tailed Student’s t-test or χ^2^ test. A P-value of <0.05 was considered statistically significant.

## Results

### Expression Profile of m5C RNA Methylation Regulators and its Correlations With Overall Survival in NPC

**Figure 1:**
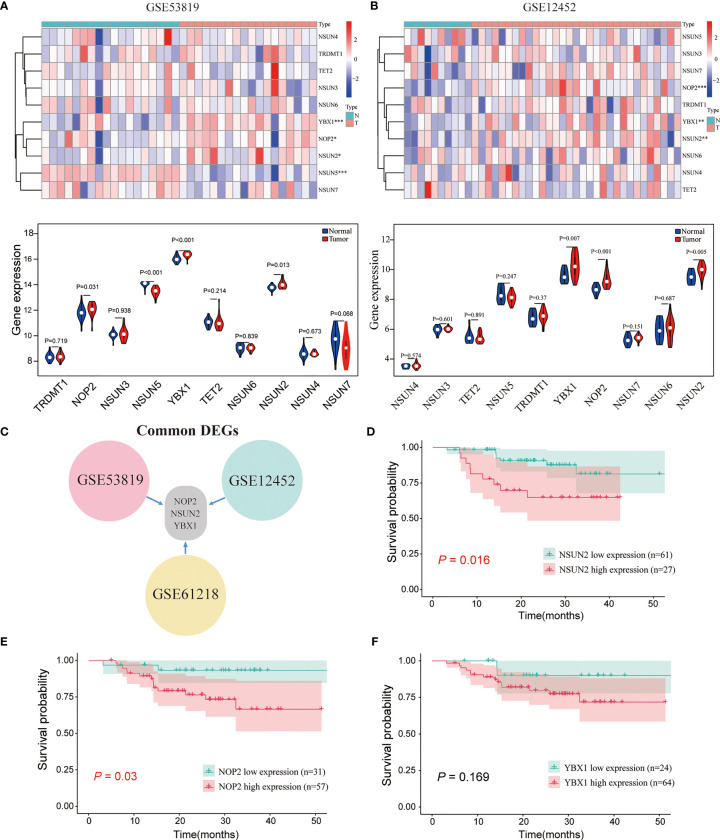
The expression landscape of m5C regulators and the correlations of their expression with the survival of patients with NPC. **(A, B)** The expression levels of m5C genes in GSE53819 and GSE12452. **(C)** The common differentially expressed m5C regulators in GSE53819, GSE12452, and GSE61218. Kaplan–Meier curves of PFS for high-and low-NSUN2 **(D)**, NOP2 **(E)**, and YBX1 **(F)** in GSE102349. *P < 0.05, **P < 0.01, and ***P < 0.001.

The common m5C regulators included 8 “writers” (NOP2, NSUN2-7, TRDMT1), 1 “eraser” (TET2), and 1 “reader” (YBX1). To investigate whether m5C regulators were aberrantly expressed in NPC, two GEO datasets (GSE53819, containing 18 NPC tissues and 18 non-cancerous nasopharyngeal tissues, and GSE12452, containing 31 NPC tissues and 10 non-cancerous nasopharyngeal tissues) were analyzed. As shown in **Figures 1A**–**C** and **S1A**, NOP2, NSUN2, and YBX1 were significantly upregulated in NPC compared with normal tissues in the three datasets. Thus, NOP2, NSUN2, and YBX1 were selected for survival analysis in GSE102349. As shown in **Figures 1D**–**F**, NOP2 and NSUN2 were associated with PFS, and high levels of NOP2 and NSUN2 predicted a poor prognosis in NPC patients in the GSE102349 cohort. Taken together, the writer of m5C regulators were mainly dysregulated in NPC, and the levels of NOP2 and NSUN2 could predict a poor prognosis for NPC patients. Considering NSUN2 is the major methyltransferase catalyzing m5C modification of mammalian mRNAs (19, 20), we chose NSUN2 for further investigation.

### NSUN2 was a Biomarker to Predict Tumor Stage, Metastasis and Poor Prognosis in NPC Patients

**Figure 2:**
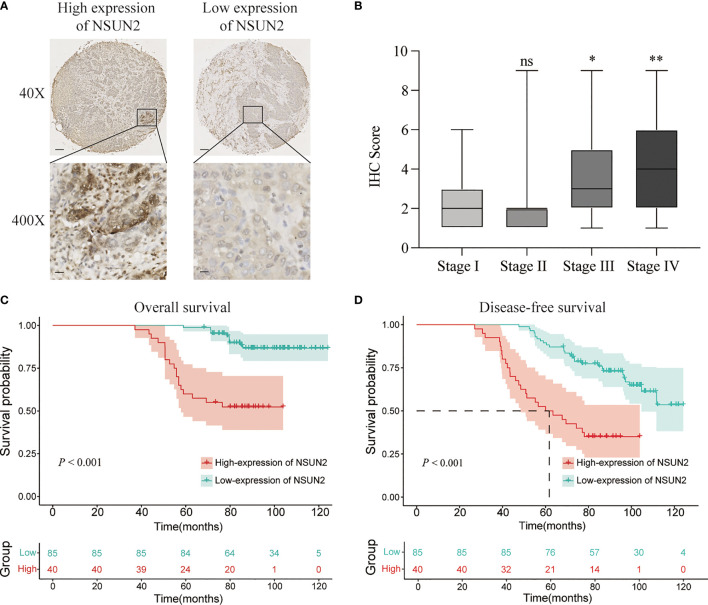
NSUN2 is expressed in the nucleus and predicts a poor prognosis of NPC patients. **(A)** Representative immunohistochemical staining of NSUN2 in NPC TMA. Brown indicates positive staining (×40: scale bar = 100 μm; ×400: scale bar = 10 μm). **(B)** Correlation between the NSUN2 expression and TNM stage. Kaplan–Meier curves for OS **(C)** and DFS **(D)** of NPC patients with NSUN2 expression. *P < 0.05 and **P < 0.01. ns, no significance.

To explore the role of NSUN2 in the development and progression of NPC, we examined the expression level of NSUN2 in tissue microarrays with 125 NPC patients by IHC. The results showed that NSUN2 was mainly expressed in the nucleus of NPC cells (**Figure 2A**), which is consistent with the localization of NSUN2 in other tumor cells. According to the intensity of the staining, we divided these patients into the NSUN2-high group (n = 40) and the NSUN2-low group (n = 85). The correlation between NSUN2 level and NPC clinicopathological features showed that the level of NSUN2 was not correlated with gender (*P* = 0.873), age (*P* = 0.293), tumor size (*P* = 0.103), but with TNM stage (*P* = 0.001), distant metastasis (*P* < 0.001), and recurrence (*P* < 0. 001) of NPC patients (**Figure 2B**) (**Table 1**), which indicated that NSUN2 might act as a precise biomarker to predict NPC progression. Kaplan–Meier survival analysis showed that patients with high NSUN2 level had a poor prognosis both in overall survival (OS) and disease-free survival (DFS) (**Figures 2C, D**). Univariate and multivariate Cox analyses showed that NSUN2 was an independent risk factor for OS (*P* =0.001, HR = 3.993, 95% CI = 1.721–9.263) and DFS (*P* < 0.003, HR =2.538, 95% CI = 1.384–4.652) in NPC (**Table 2**). In conclusion, these results indicated that NSUN2 was closely associated with malignant progression and was a key factor in the poor prognosis of NPC patients in our own cohorts.

**Table 1:** Clinicopathological features and NSUN2 in NPC.

Variables	All patients (n = 125)	Low (n = 85)	High (n = 40)	*P*-value
Gender				0.873
Female	45	31	14	
Male	80	54	26	
Age				0.293
<50	49	36	13	
≥50	76	49	27	
Tumor size				0.103
<1.2 cm	57	43	14	
≥1.2 cm	68	42	26	
TNM stage				0.001*
I + II	71	57	14	
III + IV	54	28	26	
Distant metastases				<0.001*
No	75	61	14	
Yes	50	24	26	
Recurrence				<0.001*
No	71	57	14	
Yes	54	28	26	

*Statistically significant (P <0.05).

**Table 2:** Univariate and multivariate Cox regression analyses of overall survival in patients with NPC.

Variables	OS Univariate Cox analysis		OS Multivariate Cox analysis		DFS Univariate Cox analysis		DFS Multivariate Cox analysis	
	HR (95% CI)	*P*-value	HR (95% CI)	*P*-value	HR (95% CI)	*P*-value	HR (95% CI)	*P*-value
Gender (male vs. female)	1.45 (0.95–2.20)	0.082	1.080 (0.476–2.452)	0.854	1.046 (0.593–1.846)	0.875	0.461 (0.233–0.911)	0.026
Age (≥50 vs. <50)	3.34 (1.31–9.00)	0.012*	2.600 (0.953–7.093)	0.062	2.062 (1.135–3.748)	0.018*	1.961 (1.019–3.773)	0.044*
Tumor size (≥1.2 cm vs. <1.2 cm)	1.984 (0.903–4.357)	0.088	0.684 (0.275–1.699)	0.684	2.959 (1.608–5.446)	<0.001*	0.573 (0.288–5.780)	0.113
TNM stage (III + IV vs. I + II)	25.415 (6.026–107.189)	<0.001*	7.001 (1.560–31.413)	0.011*	6.607 (3.608–12.100)	<0.001*	2.894 (1.449–5.780)	0.003*
Distant metastases (yes vs. no)	60.907 (8.268–448.653)	<0.001*	1.097 (0.125–9.620)	0.934	52.010 (18.513–146.115)	<0.001*	76.151 (22.311–269.918)	<0.001*
NSUN2 expression (high vs. low)	5.840 (2.705–12.609)	<0.001*	3.993 (1.721–9.263)	0.001*	3.938 (2.244–6.913)	<0.001*	2.538 (1.384–4.652)	0.003*

*Statistically significant (P <0.05).

### NSUN2 Promotes the Proliferation of NPC Cells *In Vitro*

**Figure 3:**
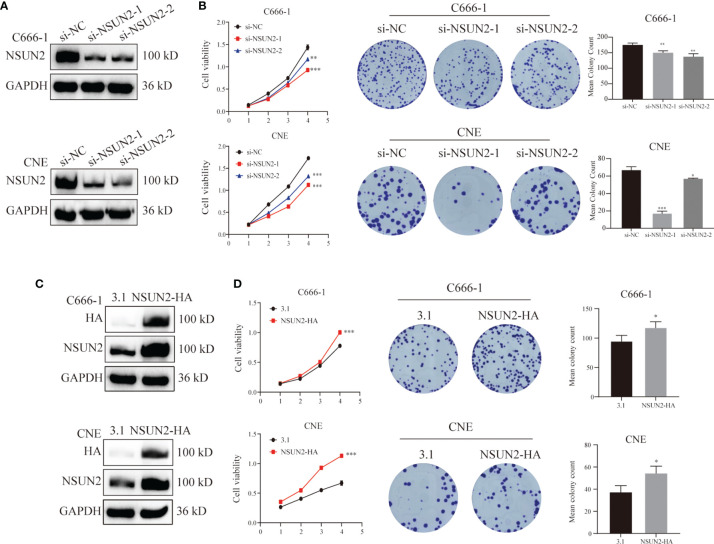
NSUN2 promote the proliferation of NPC cells. **(A, C)** Western blot analysis showed the efficiency of NSUN2 knockdown and overexpression in C666-1 and CNE cells. GAPDH as an internal reference. **(B, D)** The effects of knockout and overexpression of NSUN2 on the proliferation of NPC cells were evaluated by CCK8 and clone formation assay. *P < 0.05, **P < 0.01, and ***P < 0.001.

To further investigate the potential biological role of NSUN2 in the development of NPC, NSUN2 was either knocked down using siRNA or overexpressed using pcDNA3.1(+)/NSUN2-HA plasmid in two NPC cell lines (C666-1 and CNE). As shown in **Figures 3A, C**, the level of NSUN2 was significantly downregulated or upregulated in both NPC cell lines after transfection. Further, CCK-8 results showed that knockdown of NSUN2 could significantly inhibit the proliferation of NPC cells (**Figure 3B**), while overexpression of NSUN2 could promote the proliferation of NPC cells (**Figure 3D**). Additionally, colony formation assays were performed to determine the long-term impact of NUSN2 on NPC cell proliferation. There were more colonies formed after NSUN2 overexpression, whereas fewer colonies formed after NSUN2 knockdown. Together, these results indicate that NSUN2 promotes proliferation of NPC cells *in vitro*.

### NSUN2 Promote the Migration and Invasion of NPC Cells *In Vitro*

**Figure 4:**
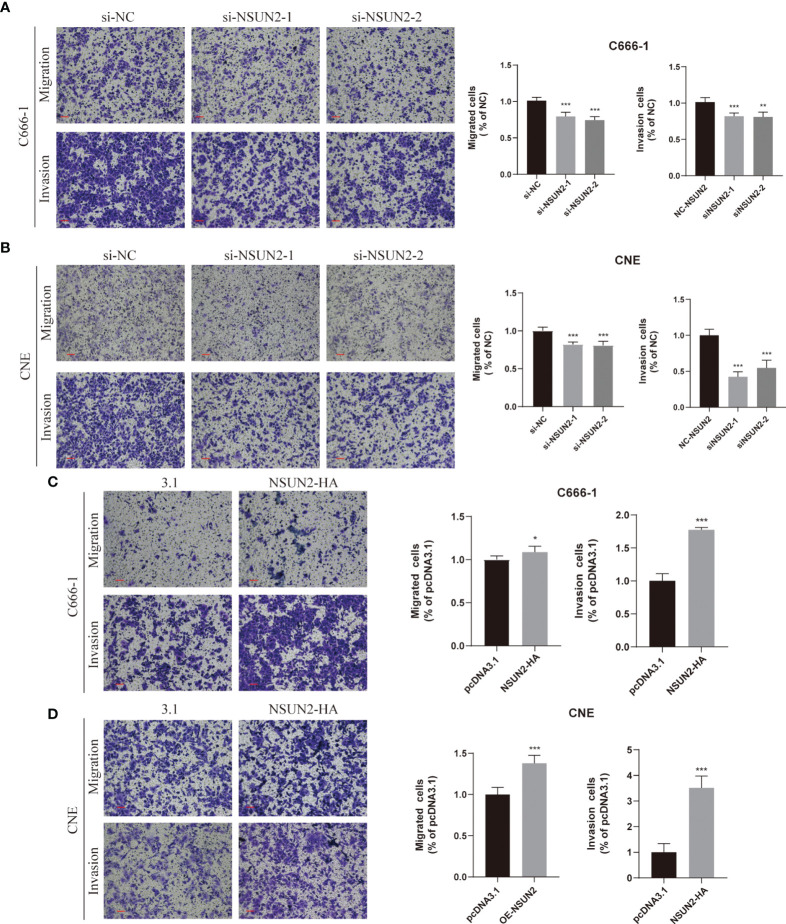
NSUN2 promotes the migration and invasion of NPC cells. Representative images of Transwell assays with C666-1 and CNE cells under NSUN2 knockdown **(A, B)** or overexpression **(C, D)**. The numbers of migrating and invading cells are presented in the right panel. Scale bar, 100 μm. *P < 0.05, **P < 0.01, and ***P < 0.001.

Because the NSUN2 level was closely related to the TNM stage and distant metastasis of NPC, we presumed that NSUN2 might prominently influence the migration and invasion ability of NPC cells. The transwell assay showed that knockdown of NSUN2 in C666-1 cells reduced the number of cells crossing the compartment and significantly reduced the invasiveness of the cells (**Figure 4A**). In contrast, overexpression of NSUN2 increased the number of cells crossing the compartment and promoted NPC cell invasion (**Figure 4C**). Additionally, similar results were observed in the CNE cell lines (**Figures 4B, D**). These results indicate that NSUN2 can promote NPC cell migration and invasion *in vitro*.

### NSUN2 has a Potential to Negatively Regulate Immune Cell Infiltration in the TME

**Figure 5:**
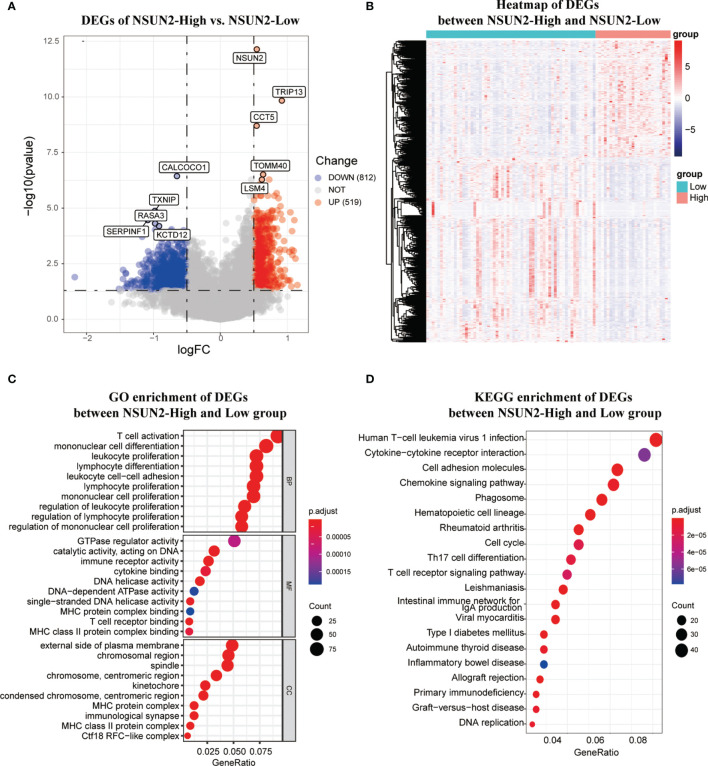
The differentially expression analysis of samples with NSUN2 high and low expression. **(A)**The volcano plot of DEGs between the NSUN2 high expression and low groups. Red points mean upregulated genes, and blue points means downregulated genes. The top 5 significant differential expressed genes were notated. **(B)** The heatmap of DEGs between the NSUN2 high and low expression groups. The GO enrichment **(C)** and KEGG enrichment **(D)** analysis of the DEGs between the NSUN2 high and low expression groups.

To further investigate the carcinogenic mechanism of NSUN2 in NPC, 88 patients in GSE102349 were divided into the NSUN2-low expression group (n = 61) and the NSUN2-high expression group (n = 27). The comparison analysis of the transcriptome between high- and low-NSUN2 expression samples was carried out. **Figures 5A, B** show that 519 genes were upregulated and 812 genes were downregulated (|logFC (fold change)| ≥0.5 and adj. *P* <0.05). The most significantly upregulated genes were *NSUN2*, *TRIP13*, *CCT5*, *TOMM40*, and *LSM4*, whereas the most significantly downregulated genes were *CALCOCO1*, *TXNIP*, *RASA3*, *SERPINF1*, and *KCTD12*. Furthermore, the 1,331 differential expression genes (DEGs) were submitted to GO and KEGG pathway analyses, and the results showed that the DEGs between the NSUN2 high- and low-groups were mainly enriched in immune response, namely, T cell activation, lymphocyte and mononuclear cell differentiation, multi-immune cell proliferation, and so on (**Figure 5C**). Additionally, KEGG enrichment analysis showed that DEGs were mainly enriched in cell adhesion molecules, chemokine signaling pathways, cell cycle, and T cell receptor signaling pathways (**Figure 5D**).

**Figure 6:**
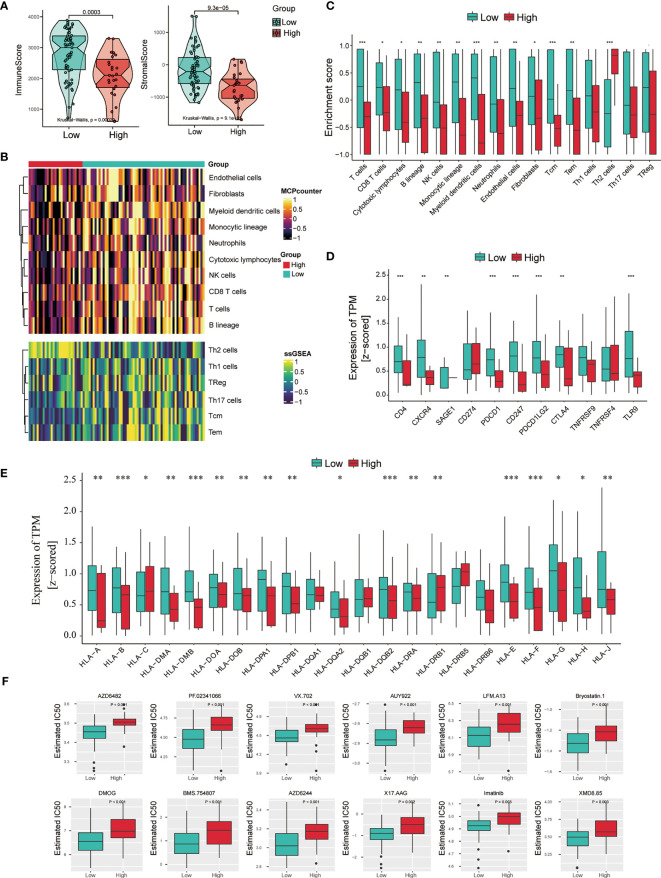
Association between immune infiltration and NSUN2 expression in NPC. **(A)** Boxplot of the ImmuneScore and StromalScore from ESTIMATE of the NSUN2 high and low expression groups. **(B)** Heatmap of the abundance of immune and stromal cell populations in the NSUN2 high and low expression groups. **(C)** Boxplot of the enrichment score of the immune cells in these two groups. **(D)** Expression level of 11 immune checkpoint genes and **(E)** HLA genes in two groups. **(F)** The sensitivity of chemotherapeutic drugs in the NSUN2 high and low expression groups. *P < 0.05, **P < 0.01, and ***P < 0.001.

These results above indicate that the level of NSUN2 may influence cell infiltration of TME in NPC. Therefore, we first assessed the immune and stromal scores in NPC and found a significant difference in immune and stromal scores between these two groups by applying the ESTIMATE algorithm (**Figure 6A**). NPC tissues with high NSUN2 levels represented lower immune and stromal scores. Next, we analyzed immune infiltration and characterized the immunologic landscape between the two groups. The MCP-counter and ssGSEA algorithms were used to calculate the abundance of 16 immune-related cell types in NPC patients (**Figure 6B**). As shown in **Figure 6C**, patients with high NSUN2 levels had a lower abundance of 12 immune cell populations (T cells, CD8 T cells, Cytotoxic lymphocytes, B lineage, NK cells, Monocytic lineage, Myeloid dendritic cells, Neutrophils, Endothelial cells, Fibroblasts, Tcm, and Tem), whereas they had a higher abundance of 1 immune cell population (Th2 cells). Considering the significant difference between the NSUN2 high- and low-expression groups, we further investigated whether NSUN2 level influences the immune checkpoint genes. As shown in **Figure 6D**, NPC patients with NSUN2 high-expression represented a lower level of seven targetable check-point genes (CD4, CXCR4, PDCD1, CD247, PDCD1LG2, CTLA4, and TLR9) than the NSUN2 low-expression group. Additionally, we found that most human leukocyte antigen (HLA) genes were also expressed at higher levels in the NSUN2 low-expression group, which indicated a potential association between the NSUN2 expression level and immunotherapy efficacy in NPC patients (**Figure 6E**). Furthermore, the R package pRRophetic was used to analyze the response to chemotherapy in GSE102349 NPC patients with the NSUN2 low- and high-expressions. We found that 55 chemotherapeutic drugs showed a significant difference in estimated IC50 between these two groups, and patients in the NSUN2-low group displayed higher sensitivity to most of the chemotherapeutic drugs than those in the NSUN2-high group (**Figure 6F**). At present, cisplatin, 5-fluorouracil, and paclitaxel are first-line chemotherapy drugs for the clinical treatment of NPC (21). Moreover, studies have found that new chemotherapy drugs such as trametinib, docetaxel, and oxaliplatin can inhibit the growth of NPC cells, providing an experimental basis for the clinical treatment of NPC patients (22–24). We conducted an *in vitro* drug sensitivity test on NPC cells with different levels of NSUN2, and the results showed that the cells with a high level of NSUN2 were more resistant to oxaliplatin than those with low levels (**Supplementary Figure 1B**). Taken together, these results reveal that NSUN2 has the potential to negatively regulate immune cell infiltration in TME and is closely related to chemotherapy resistance.

## Discussion

Different from other HNSC, NPC has its own unique biological characteristics, epidemiology (25), and etiology (26). Approximately 70% of all NPC patients are locally advanced at initial diagnosis because of the lack of obvious symptoms in the early period (27). Although treatments based on radiotherapy and chemotherapy have significantly improved for treating non-metastatic NPC (28), distal metastasis remains the major cause of death in NPC (29). Therefore, it is critical to find reliable tumor markers for early diagnosis and molecular targeted therapy for NPC. In this study, we demonstrated that NSUN2 was highly expressed in NPC and might predict a poor prognosis because it was closely correlated with the tumor stage and distant metastasis in the GEO database and our own cohorts. Moreover, NSUN2 promotes the malignancy of NPC cells *in vitro* and might negatively regulate the infiltration of immune cells into TME.

Increasing evidence has demonstrated that post-transcriptional modifications of RNA, such as m6A and m5C, have played a vital role in the progression of multiple cancers. Previous studies have reported that regulators of these RNA modifications dysregulate expression in most cancers and mediate multiple oncogenic pathways. However, few studies have reported the expression level and the biological role of m5C regulators in head and neck malignancies, especially NPC. Here, we displayed the landscape of the expression levels of m5C regulators in NPC and normal tissues based on the GSE53819, GSE12452, and GSE61218 datasets. By taking the intersection of three datasets, NOP2, NSUN2, and YBX1 were all upregulated in NPC compared with normal tissues. Next, we analyzed the survival information of the three genes in the NPC-based GSE102349 dataset and found only NOP2 and NSUN2 were correlated with poor survival of NPC patients, among which NSUN2 had more impact on prognosis. Moreover, NSUN2 is also the main m5C modification methyltransferase of mammalian mRNA. Therefore, we anchored NSUN2 as our target gene for further study of NPC.

As a writer of m5C RNA modification, NSUN2 has been reported to be closely related to the spliceosome, RNA degradation, cell cycle, and RNA polymerase (30). Accumulated evidence has demonstrated that NSUN2 is overexpressed in various tumors such as low-grade glioma (31), gastric cancer (27), triple-negative breast cancer (30), and so on. In this study, NSUN2 was highly expressed in NPC tissues, which was consistent with other tumors reported. Moreover, an increasing number of studies have confirmed that NSUN2 plays a carcinogenic role in tumorigenesis. For example, NSUN2 methylation can promote ATX exudation, enhance mRNA transcription, and influence the migration of glioma cells U87 through the NSUN2–ATX–LPA axis (32, 33). Mei et al. found that NSUN2 acts as an oncogene to inhibit p57 in an m5C-dependent manner in gastric cancer (27). Another study suggested that NSUN2 has a high mutation rate in gastric and esophageal cancers and has more protein alteration sites (34). In hepatocellular carcinoma, NSUN2 affects the biological function of hepatocellular carcinoma cells and methylates lncRNA H19RNA and methylated H19 recruits oncogene G3BP1 to promote the occurrence and development of tumors (35). Here, we demonstrated that NSUN2 was closely associated with poor prognosis and advanced pathological stages of NPC by IHC in our cohort, and future studies with large samples must confirm our findings. Meanwhile, NSUN2 could significantly promote the carcinogenicity of NPC cells because we observed that NSUN2 enhanced the proliferation and colony formation of NPC cells and promoted the migration and invasion of NPC cells, followed previous studies on other tumors. To explore the mechanism of NSUN2 expression and malignant progression and poor prognosis of NPC, we divided NPC patients into the NSUN2-low and NSUN2-high expression groups based on the group of Kaplan–Meier analysis in GSE102349. We found a significant difference in the transcriptome expression between these two groups. For example, common oncogenes, such as TRIP13 (36) were highly expressed in the NSUN2-high expression group, whereas the recognized tumor suppressor, TXNIP (37) was mainly expressed in the NSUN2-low expression group. Surprisingly, the GO and KEGG enrichment analyses showed that the DEGs between the NSUN2-high and -low groups were mainly enriched in multi-immune cell activation, differentiation, and proliferation, which indicated that NSUN2 is closely related to the infiltration of immune cells into NPC tissues.

Tumor progression was previously considered to be involved only in genetic and epigenetic variations of tumor cells (38). Recently, various studies showed that other cells in the TME such as immune cells, fibroblasts, inflammatory cells, and glial cells, also play essential roles in tumor progression, immune escape, and even drug resistance (39). Immunotherapy based on the TME of tumors has also gained widespread attention in cancer treatment (40, 41). NPC is a heterogeneous epithelial tumor characterized by EBV infection and severe lymphocyte infiltration (42). These special characteristics suggest an important role for TME in its progression. Therefore, we assessed the proportion of immune and stromal components in the NSUN2-high and low groups and found that both the ImmuneScore and StromalScore were significantly higher in the NSUN2-low expression group than in the NSUN2-high group, which implied numerous immune components in the NSUN2-low group. Additionally, we analyzed the degree of 16 immune-related cell type infiltration in the two NPC groups. Most of these immune cells were consistently highly infiltrated in the NSUN2-low group. Studies have shown that the degree of immune infiltration is related to the prognosis of patients, and a high level of immune infiltration generally predicts a better prognosis (43). Meanwhile, immune infiltration is closely associated with the response of tumors to immunotherapy (44). Predicting the response of immunotherapy based on the infiltration characteristics of TME is a crucial step to improving the success rate of existing immunotherapy and developing new immunotherapy strategies. For example, immunotherapies, such as immune checkpoint blockade against PD-1 and CTLA-4, have become a promising strategy for treating various malignancies (45, 46). Hence, we evaluated the expression level of common immune checkpoint genes and found that most of these genes were highly expressed in the NSUN2-low group, suggesting that it responds positively to these treatments. Furthermore, as an independent factor of tumor-associated antigen presentation, the HLA family plays an important role in anti-tumor immune response and neoplastic progression (47). Theoretically, diverse HLA with distinct binding specificity lead to the presentation of diverse epitopes by different individuals which increases the possibility of presenting more immunogenic antigens, and increases the possibility of benefiting from ICBs (47, 48). Here, we showed that most HLA genes were highly expressed in the NSUN2-low groups, which also indicated that NSUN2 might be negatively associated with the efficacy of immunotherapy. Chemotherapy is one of the main treatments for NPC. Clinically, gemcitabine combined with cisplatin is an effective combination for treating patients with locoregionally advanced NPC (49, 50). However, chemotherapy resistance invariably develops and results in cancer recurrence and malignant progression. Here, we estimated the relationship between NSUN2 expression and the sensitivity of chemotherapy and found that NPC cell lines with lower NSUN2 expression are more sensitive to most chemotherapy. For example, AZD6482 is an inhibitor of PI3Kβ and exerts an anti-tumor role by inhibiting the proliferation and inducing apoptosis of human glioma cells (51). PF.02341066, a c-Met inhibitor, has been reported to be one of the effective therapeutic strategies for patients with lung cancer (52). We found that NPC cell lines with low expression of NSUN2 had higher sensitivity to AZD6482 and PF.02341066, which may provide a new choice for selecting chemotherapy drugs for NPC patients. Taken together, the above results suggest that NSUN2 has the potential to negatively regulate immune cell infiltration in the TME and was negatively correlated with the sensitivity of immunotherapy and chemotherapy drugs for NPC. Therefore, NSUN2 may act as a predictive biomarker of drug resistance in clinical chemotherapy and immunotherapy.

In summary, we found that NSUN2 was highly expressed in NPC tissues and the level of NSUN2 was closely correlated with tumor stage and distant metastasis. It might serve as a poor prognosis for NPC patients. Furthermore, we confirmed that NSUN2 significantly promoted the malignant phenotype of NPC cells *in vitro*. Additionally, NSUN2 might be negatively associated with multi-immune cell proliferation and infiltration in TME. Based on the level of immune checkpoint genes and the results of drug sensitivity tests *in vitro*, we speculated that patients with high levels of NSUN2 showed worse benefits from chemotherapy and immunotherapy. Overall, NSUN2 might act as an oncogene and predict poor prognosis and poor drug sensitivity in NPC patients.

## Data Availability Statement

The original contributions presented in the study are included in the article/**Supplementary Material**. Further inquiries can be directed to the corresponding author.

## Author Contributions

SZ, XX, and KZ designed the overall study and revised the paper. XT, YX, and YH drafted the manuscript and performed the experiments. XT, YYH, and HL participated in the data collection and analysis. XT and HW acquired the data and material support. All authors listed have made a substantial, direct, and intellectual contribution to the work and approved it for publication.

## Funding

This research was supported by the Zhejiang Provincial Natural Science Foundation of China under Grant No. LY22H160015.

## Conflict of Interest

The authors declare that the research was conducted in the absence of any commercial or financial relationships that could be construed as a potential conflict of interest.

## Publisher’s Note

All claims expressed in this article are solely those of the authors and do not necessarily represent those of their affiliated organizations, or those of the publisher, the editors and the reviewers. Any product that may be evaluated in this article, or claim that may be made by its manufacturer, is not guaranteed or endorsed by the publisher.
