# Supplementary material for: NSUN2 Promotes Tumor Progression and Regulates Immune Infiltration in Nasopharyngeal Carcinoma
Source: Front Oncol. 2022 Apr 29;12:788801. doi: 10.3389/fonc.2022.788801 (PMC9099203; doi:10.3389/fonc.2022.788801)
Supplement: Supplementary file 1 [file DataSheet_1.docx]

**Supplementary information**

**Methods**

**Generation of NSUN2-knockout NPC cell lines**

NSUN2-KO CNE cell lines were constructed using the CRISPR–Cas9 gene-editing system. Lentiviruses containing Cas9-guide RNA targeting sequences (5′-TGTTCTCCTTGACGATCTCG-3′) were designed and synthesized by HANBIO (Shanghai, China). Lentivirus infection was performed on CNE cells at 80% confluency, with a multiplicity of infection (MOI) of 60. The cells were selected after culture for one week in a medium containing 2 μg/ml puromycin (MCE, Shanghai, China). The knockout efficiency was determined by western blotting.

**Drug sensitivity test**

CNE cells in good growth condition were digested and counted, and then inoculated into 96-well plates at a density of 3000 cells/well, and placed in the incubator overnight. After the media was discarded, cells were continued to be cultured in complete media containing different drug concentrations. After 48h, cell viability was detected by CCK8 reagent.

**Supplementary Figure 1.** (A) The expression levels of m5C genes in GSE61218. (B) Generation of NSUN2-knockout CNE cell line and the inhibition efficiency of Oxaliplatin in CNE cell line. **P* < 0.05, ***P* < 0.01, and ****P* < 0.001.
