# Supplementary figures and images for: NSUN2 Promotes Tumor Progression and Regulates Immune Infiltration in Nasopharyngeal Carcinoma
Source: Front Oncol. 2022 Apr 29;12:788801. doi: 10.3389/fonc.2022.788801 (PMC9099203; doi:10.3389/fonc.2022.788801)

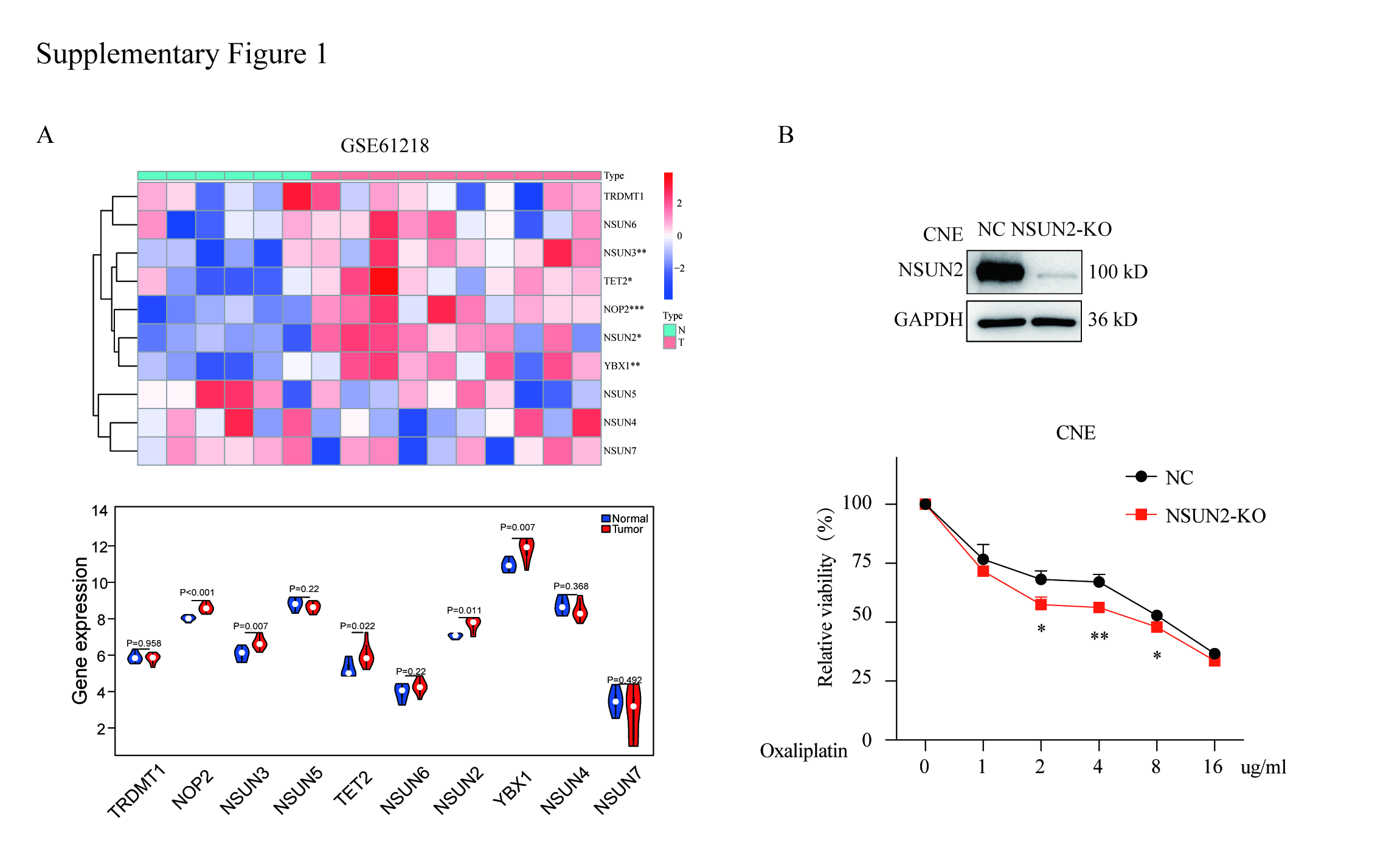

Supplement: Supplementary file 2 [file Image_1.tif]
